# Decreased susceptibility to ciprofloxacin in Salmonella enterica serotype typhi, United Kingdom.

**DOI:** 10.3201/eid0703.010315

**Published:** 2001

**Authors:** E J Threlfall, L R Ward

**Affiliations:** Central Public Health Laboratory, London, United Kingdom. jthrelfall@phls.nhs.uk

## Abstract

In 1999, 23% of Salmonella enterica serotype Typhi isolates from patients in the United Kingdom exhibited decreased susceptibility to ciprofloxacin (MIC 0.25-1.0 mg/L); more than half were also resistant to chloramphenicol, ampicillin, and trimethoprim. Increasing numbers of treatment failures have been noted. Most infections have been in patients with a recent history of travel to India and Pakistan.


### Decreased Susceptibility to Ciprofloxacin in *Salmonella enterica* serotype Typhi, United Kingdom

**E. John Threlfall and Linda R. Ward**
Central Public Health Laboratory, London, United Kingdom

In 1999, 23% of *Salmonella enterica* serotype Typhi isolates from patients in the United Kingdom exhibited decreased susceptibility to ciprofloxacin (MIC 0.25-1.0 mg/L); more than half were also resistant to chloramphenicol, ampicillin, and trimethoprim. Increasing numbers of treatment failures have been noted. Most infections have been in patients with a recent history of travel to India and Pakistan.

*Salmonella enterica* serotype Typhi is endemic in developing countries in Africa, South and Central America, and the Indian subcontinent, with an estimated incidence of 33 million cases each year (1). By contrast, in developed countries such as the United Kingdom or the USA, incidence is much lower, and most cases are in travelers returning from endemic areas. For example, 150 to 300 cases occur each year in the U.K., at least 70% in patients with a history of recent foreign travel.

For patients with typhoid fever, administration of an effective antibiotic should begin as soon as clinical diagnosis is made, without recourse to results of antimicrobial sensitivity tests. From 1948 to the mid-1970s, chloramphenicol was the first-line drug of choice, and in developed countries its use resulted in a reduction in mortality rates from 10% to <2%. After extensive outbreaks of typhoid fever occurred in Mexico and India in the early and mid-1970s, in which epidemic strains were resistant to chloramphenicol (2,3), the efficacy of this antimicrobial agent was in doubt.

Alternative drugs for typhoid fever are ampicillin and trimethoprim. However, following outbreaks in the Indian subcontinent, the Arabian Gulf, the Philippines, and South Africa in the late 1980s and early 1990s, in which causative strains were resistant to ampicillin and trimethoprim in addition to chloramphenicol, the efficacy of these antimicrobial agents has also been impaired (4).

The Laboratory of Enteric Pathogens of the Public Health Laboratory Service of England and Wales is the reference center in the U.K. for strains of *S.* Typhi. Strains are identified by Vi-phage typing; all strains are tested with an agar dilution method for resistance to a panel of antimicrobial drugs. The final plate concentrations for selected antimicrobial drugs were chloramphenicol 8 mg/L, ampicillin 8 mg/L, trimethoprim 2 mg/L, nalidixic acid 16 mg/L, ciprofloxacin 0.125 mg/L, ceftriaxone 1 mg/L, and cefotaxime 1 mg/L.

For isolates resistant to ciprofloxacin at 0.125 mg/L, full MICs are determined either by incorporating doubled concentrations of the antimicrobial agent into the agar substrate or by E-test. All isolates resistant to ciprofloxacin at 0.125 mg/L were also resistant to nalidixic acid at 16 mg/L. In contrast, isolates sensitive to nalidixic acid at 16 mg/L had MICs to ciprofloxacin of <0.025 mg/L. All strains with resistance to chloramphenicol, ampicillin, trimethoprim, or nalidixic acid/ciprofloxacin were tested for the ability to transfer these resistances to a drug-sensitive strain of *Escherichia coli* K12. Resultant resistance plasmids were characterized by incompatibility grouping and agarose gel electrophoresis after extraction of plasmid DNA from donor strains of *S.* Typhi and recipient strains of *E. coli* K12.

From 1978 to 1985, resistance to chloramphenicol was identified in 11 (0.47%) of 2,356 strains studied (5); therefore, chloramphenicol remained the first-line drug for typhoid fever before results of laboratory sensitivity tests became available. From 1986 to 1989, chloramphenicol resistance increased threefold: 12 (1.5%) of 790 isolates were resistant. However, this increase was not considered sufficient to change recommendations about therapy. In 1990, there was a dramatic change, with 20% of 248 isolates resistant to chloramphenicol; most were also resistant to ampicillin and trimethoprim (6). In 1991, because of this increased chloramphenicol resistance, ciprofloxacin was recommended as an alternative for patients with a history of recent travel to epidemic areas (7).

From 1990 to 1999, 151 to 291 (mean 210) patients per year in the U.K. had typhoid fever. The incidence of multidrug resistance (MDR) to chloramphenicol, ampicillin, and trimethoprim increased from 21% in 1991 to 36% in 1994, declined to 13% in 1997, and then increased to 26% in 1999 (Table 1). More than 90% of patients infected with MDR strains had recently returned from the Indian subcontinent, particularly Pakistan and India. The reasons for the decline in MDR strains in 1997 followed by the return to 1996 levels in 1998 and 1999 are not known but may be related to changes in climatic conditions in the Indian subcontinent in the mid-1990s, followed by reestablishment of MDR strains in different parts of India in the late 1990s. Epidemiologic investigations to test these hypotheses are in progress.

In the early 1990s, the most common Vi-phage type in MDR strains was phage type M1, and almost all patients

Address for correspondence: E. John Threlfall, Laboratory of Enteric Pathogens, Central Public Health Laboratory, 61 Colindale Avenue, London NW9 5HT, U.K.; fax: + 44 0208 905 9929; e-mail: jthrelfall@phls.nhs.uk

Table 1. Incidence of multidrug resistance and decreased susceptibility to ciprofloxacin in *Salmonella enterica* serotype Typhi, U.K., 1990–1999

| Year | Strains[a] | MDR[b] no. (%) | Decreased suscepti-bility to $CP_L$ no. (%) | $CP_L$ strains also resistant to: C | A | Tm | Ct | Cf |
|---|---|---|---|---|---|---|---|---|
| 1990 | 248 | 50 (20) | 0 (0) | 0 | 0 | 0 | 0 | 0 |
| 1991 | 226 | 48 (21) | 2 (0.9) | 1 | 1 | 1 | 0 | 0 |
| 1992 | 204 | 49 (24) | 1 (0.5) | 0 | 0 | 0 | 0 | 0 |
| 1993 | 194 | 49 (25) | 1 (0.5) | 1 | 1 | 1 | 0 | 0 |
| 1994 | 259 | 94 (36) | 5 (2) | 5 | 5 | 5 | 0 | 0 |
| 1995 | 291 | 100 (34) | 8 (3) | 5 | 5 | 5 | 0 | 0 |
| 1996 | 210 | 52 (25) | 11 (7) | 7 | 7 | 7 | 0 | 0 |
| 1997 | 174 | 22 (13) | 9 (5) | 6 | 6 | 6 | 0 | 0 |
| 1998 | 151 | 34 (23) | 32 (21) | 19 | 19 | 19 | 0 | 0 |
| 1999 | 179 | 47 (26) | 42 (23) | 25 | 25 | 25 | 0 | 0 |

[a]Strains referred to Laboratory of Enteric Pathogens.
[b]MDR = multidrug resistant (to chloramphenicol, ampicillin, and trimethoprim). Resistance symbols: C, chloramphenicol; A, ampicillin; Tm, trimethoprim; $CP_L$, ciprofloxacin (MIC 0.25-1.0 mg/L); Ct, ceftriaxone; Cf, cefotaxime. Percentages of total isolates in parentheses.

infected with strains of this phage type had acquired the infections in Pakistan. The last isolations of MDR phage type M1 in the U.K. were in 1994 (4). Since 1993, the most common MDR phage type has been E1. Most patients infected with MDR strains of phage type E1 had acquired the infections in India or Pakistan. However, infections were also recorded in patients returning from Bangladesh, Sri Lanka, and Afghanistan. Regardless of phage type, in all MDR strains resistance to chloramphenicol, ampicillin, and trimethoprim has been encoded by plasmids of approximately 100 megadaltons belonging to the $H_1$ incompatibility group.

In 1991, a strain of *S.* Typhi with plasmid-encoded resistance to chloramphenicol, ampicillin, and trimethoprim and with chromosomally encoded resistance to nalidixic acid (MIC 512 mg/L) was isolated from a 1-year-old child who had recently returned from India. The strain also showed a marked decrease in sensitivity to ciprofloxacin (MIC 0.6 mg/L). The patient did not respond to treatment with ciprofloxacin despite serum levels of 1.5 mg/L. In 1995, 8 (3%) of 291 isolates showed decreased sensitivity to ciprofloxacin (MICs 0.38–0.75 mg/L by E-test); 5 were also resistant to chloramphenicol, ampicillin, and trimethoprim. In 1998, 32 (21%) of 151 strains exhibited decreased susceptibility to ciprofloxacin. One patient, a 65-year-old woman who returned from India infected with a strain of phage type E1 (MIC to ciprofloxacin of 1.0 mg/L) did not respond to twice a day treatment with ciprofloxacin, 400 mg intravenously. After 5 days, treatment was changed to amoxicillin and ceftriaxone. Within 3 days, the patient's condition improved, and after a further 5 days she was apyrexial (8).

*S.* Typhi with decreased susceptibility to ciprofloxacin increased to 23% in the U.K. in 1999 (Table 1). All strains with decreased sensitivity to ciprofloxacin were also resistant to nalidixic acid (MIC 512 mg/L). The predominant phage types have been E1 (81% of cases) and E9 (4%). However, strains of phage types C2, E7, M1, untypeable Vi (UVS), and Vi-negative have also been identified. Most patients had recently returned from India or Pakistan. However, in 1998 and 1999, strains with decreased susceptibility to ciprofloxacin were also isolated from travelers returning from Sri Lanka, Nepal, Bangladesh, and Thailand (Table 2). Furthermore, in both years >50% of isolates with decreased susceptibility to ciprofloxacin were also MDR (Table 1). In 1999, at least 10 patients infected with strains with decreased susceptibility to ciprofloxacin did not respond to treatment with fluoroquinolone antimicrobials. In such cases, ceftriaxone was the most frequently used alternative. In contrast to resistance to chloramphenicol, ampicillin, and trimethoprim, resistance to ciprofloxacin has been chromosomally encoded in all isolates with decreased sensitivity to this antimicrobial agent.

Since 1993, strains of *S.* Typhi with decreased susceptibility to ciprofloxacin have been isolated with increasing frequency in Vietnam (9). In 1997, >6,000 cases occurred in an extensive epidemic in Tajikistan of nalidixic acid-resistant *S.* Typhi with decreased susceptibility to ciprofloxacin (10). The epidemic strain was untypeable with the Vi typing phages but had a pulsed-field profile indistinguishable from that of isolates of MDR Vi-phage type E1 from patients infected in India (11). In both Vietnam and Tajikistan, treatment failures with fluoroquinolone antibiotics have been noted.

The accepted British Society for Antimicrobial Chemotherapy and National Committee for Clinical Laboratory Standards' zone size equivalents for resistance to ciprofloxacin in disc diffusion tests are 2 mg/L and 4 mg/L, respectively, for Enterobacteriaceae. However, testing for resistance at these levels could result in decreased susceptibilities not being detected. As all strains with decreased susceptibility to ciprofloxacin have also been resistant to nalidixic acid, we suggest that the latter antimicrobial agent be included in the panel of drugs used for sensitivity testing. If resistance to nalidixic acid is detected, full MICs to ciprofloxacin should be performed in the event of treatment failure.

Table 2. Phage types in *Salmonella* Typhi isolates with decreased susceptibility to ciprofloxacin ($C_{pL}$), United Kingdom, 1991–1999

| Year | $C_{pL}$ | Phage types: E1 | M1 | E9 | Others | Country of origin (no.) |
|---|---|---|---|---|---|---|
| 1991 | 2 | 2 | 0 | 0 | 0 | India (1), Nepal (1) |
| 1992 | 1 | 0 | 0 | 0 | 1 (B2) | India (1) |
| 1993 | 1 | 0 | 0 | 0 | 1 (E14) | Bangladesh (1) |
| 1994 | 5 | 5 | 0 | 0 | 0 | India (3), Nepal (1), Bangladesh (1) |
| 1995 | 8 | 6 | 1 | 0 | 1 (D1) | India (4), Pakistan (3), NS[a] (1) |
| 1996 | 11 | 11 | 0 | 0 | 0 | India (9), Pakistan (2) |
| 1997 | 9 | 8 | 0 | 0 | 1(A) | India (5), Pakistan (2), Nepal (1), NS (1) |
| 1998 | 32 | 23 | 0 | 6 | 3 (G2,2;O,1) | Pakistan (14), India (9), Sri Lanka (1), Bangladesh (1), NS (7) |
| 1999 | 42 | 27 | 1 | 3 | 11 (UVS, 7; E7, 1; C2, 1; Vi-negative, 1) | India (31), Pakistan (6), Bangladesh (1), Thailand (1), NS (3) |

[a]NS = details of travel itinerary not provided; UVS = untypeable with the Vi typing phages.

Our findings suggest that strains of *S.* Typhi with decreased sensitivity to ciprofloxacin are now endemic in several countries in the Indian subcontinent and that such strains are increasing in travelers returning to the U.K. Despite the low level of resistance, treatment failures are being increasingly noted. In such cases, possible alternatives such as ceftriaxone or cefotaxime could be considered. In this respect, it is reassuring that all strains of *S.* Typhi so far tested were sensitive to these antimicrobial drugs.

Dr. Threlfall is head of the Antibiotic Resistance and Molecular Epidemiology Laboratory at the Central Public Health Laboratory of the Public Health Laboratory Service of England and Wales.

Mrs. Ward is head of the Salmonella Reference Unit in the Public Health Laboratory Service of England and Wales.

### References

1. Ivanoff B. Typhoid fever: global situation and WHO recommendations. Southeast Asian J Trop Med Public Health 1995; 26(Suppl 2):1-6.
2. Anderson ES, Smith HR. Chloramphenicol resistance in the typhoid bacillus. BMJ 1972;3:329-31.
3. Paniker CKJ, Vilma KN. Transferable chloramphenicol resistance in *Salmonella typhi*. Nature 1972;239:109-10.
4. Rowe B, Ward LR, Threlfall EJ. Multidrug-resistant *Salmonella typhi*: a worldwide epidemic. Clin Infect Dis 1997;24(Suppl 1): S106-9.
5. Rowe B, Threlfall EJ, Ward LR. Does chloramphenicol remain the drug of choice for typhoid? Epidemiol Infect 1987;98:379-83.
6. Rowe B, Ward LR, Threlfall EJ. Spread of multiresistant *Salmonella typhi*. Lancet 1990;336:1065-6.
7. Rowe B, Ward LR, Threlfall EJ. Treatment of multiresistant typhoid fever. Lancet 1991;337:1422.
8. Threlfall EJ, Ward LR, Skinner JA, Smith HR, Lacey S. Ciprofloxacin-resistant *Salmonella typhi* and treatment failure. Lancet 1999;353:1590-1.
9. Parry C, Wain J, Chinh NT, Vinh Ha, Farrar JJ. Quinolone-resistant *Salmonella typhi* in Vietnam. Lancet 1998;351:1289.
10. Murdoch DA, Banatvala NA, Bone A, Shoismatulloev BI, Ward LR, Threlfall EJ, et al. Epidemic ciprofloxacin-resistant *Salmonella typhi* in Tajikistan. Lancet 1998;351:339.
11. Hampton MD, Ward LR, Rowe B, Threlfall EJ. Molecular fingerprinting of multidrug-resistant *Salmonella enterica* serotype Typhi. Emerg Infect Dis 1998;4:317-20.
